# Examining disparities in large‐scale patient‐reported data capture using digital tools among cancer patients at clinical intake

**DOI:** 10.1002/cam4.6459

**Published:** 2023-08-18

**Authors:** Dana E. Rollison, Brian D. Gonzalez, Kea Turner, Heather S. L. Jim, Yayi Zhao, Rossybelle P. Amorrortu, Rachel Howard, Kavita M. Ghia, Bryan Ngo, Phillip Reisman, Colin Moore, Randa Perkins, Robert J. Keenan, David A. Sallman, Cristina M. Naso, Edmondo J. Robinson, Susan T. Vadaparampil, Vani N. Simmons, Matthew B. Schabath, Scott M. Gilbert

**Affiliations:** ^1^ Department of Cancer Epidemiology Moffitt Cancer Center Tampa Florida USA; ^2^ Department of Health Outcomes and Behavior Moffitt Cancer Center Tampa Florida USA; ^3^ Department of Health Informatics Moffitt Cancer Center Tampa Florida USA; ^4^ Collaborative Data Services Core, Moffitt Cancer Center Tampa Florida USA; ^5^ Department of Business Intelligence and Analytics Moffitt Cancer Center Tampa Florida USA; ^6^ Department of Clinical Informatics Moffitt Cancer Center Tampa Florida USA; ^7^ Department of Thoracic Oncology Moffitt Cancer Center Tampa Florida USA; ^8^ Department of Malignant Hematology Moffitt Cancer Center Tampa Florida USA; ^9^ Department of Virtual Health Moffitt Cancer Center Tampa Florida USA; ^10^ Center for Digital Health Moffitt Cancer Center Tampa Florida USA; ^11^ Department of Internal and Hospital Medicine Moffitt Cancer Center Tampa Florida USA; ^12^ Department of Genitourinary Oncology Moffitt Cancer Center Tampa Florida USA

**Keywords:** adults, healthcare disparities, neoplasms, population groups, surveys and questionnaires

## Abstract

**Background:**

Patient‐reported data can improve quality of healthcare delivery and patient outcomes. Moffitt Cancer Center (“Moffitt”) administers the Electronic Patient Questionnaire (EPQ) to collect data on demographics, including sexual orientation and gender identity (SOGI), medical history, cancer risk factors, and quality of life. Here we investigated differences in EPQ completion by demographic and cancer characteristics.

**Methods:**

An analysis including 146,142 new adult patients at Moffitt in 2009–2020 was conducted using scheduling, EPQ and cancer registry data. EPQ completion was described by calendar year and demographics. Logistic regression was used to estimate associations between demographic/cancer characteristics and EPQ completion. More recently collected information on SOGI were described.

**Results:**

Patient portal usage (81%) and EPQ completion rates (79%) were consistently high since 2014. Among patients in the cancer registry, females were more likely to complete the EPQ than males (odds ratio [OR] = 1.17, 95% confidence interval [CI] = 1.14–1.20). Patients ages 18–64 years were more likely to complete the EPQ than patients aged ≥65. Lower EPQ completion rates were observed among Black or African American patients (OR = 0.59, 95% CI = 0.56–0.63) as compared to Whites and among patients whose preferred language was Spanish (OR = 0.40, 95% CI = 0.36–0.44) or another language as compared to English. Furthermore, patients with localized (OR = 1.16, 95% CI = 1.12–1.19) or regional (OR = 1.16, 95% CI = 1.12–1.20) cancer were more likely to complete the EPQ compared to those with metastatic disease. Less than 3% of patients self‐identified as being lesbian, gay, or bisexual and <0.1% self‐identified as transgender, genderqueer, or other.

**Conclusions:**

EPQ completion rates differed across demographics highlighting opportunities for targeted process improvement. Healthcare organizations should evaluate data acquisition methods to identify potential disparities in data completeness that can impact quality of clinical care and generalizability of self‐reported data.

## 1 BACKGROUND

Healthcare providers routinely use new patient (NP) intake questionnaires to collect data on patient demographics, personal and family medical history, social needs, and current symptoms.^1^, ^2^, ^3^ Cancer patients often have unmet needs, such as unmanaged symptoms or barriers to healthcare, that can be identified through these questionnaires. Prior studies demonstrate that collecting patient‐reported data can improve health outcomes, reduce adverse events, and expand access to care.^4^, ^5^, ^6^ Specifically, some cancer centers have developed enterprise‐wide^7^, ^8^, ^9^ or clinic‐specific^10^ intake forms to collect pertinent patient‐reported data prior to a patient's first visit. Equitable participation in these data collection efforts is essential for ensuring that all patients receive patient‐centered care. However, to the best of our knowledge, no information is available on how the completeness of these data vary demographic factors among cancer patients.

H. Lee Moffitt Cancer Center & Research Institute (“Moffitt”), a National Cancer Institute‐Designated Comprehensive Cancer Center in Tampa, Florida delivers multidisciplinary cancer care through disease‐based clinics that historically administered paper questionnaires specific to each clinic. Paper‐based questionnaires included overlapping questions across clinics, contributing to patient burden and inefficiencies, and limited use of patient‐reported data for clinical decision support, quality improvement and research. In 2009, Moffitt began administering, to all NPs, the Electronic Patient Questionnaire (EPQ), a clinical intake form for the ascertainment of baseline demographic and clinical data. The goal of the EPQ was to scale data acquisition across thousands of patients simultaneously, ensure EPQ data were available to healthcare providers directly in the electronic medical record, reduce patient burden through harmonization of questions across clinics and more rapidly introduce changes to accommodate evolving clinical and operational needs. Primarily deployed through a patient portal which most patients access days before their first visit, the EPQ can be completed in multiple sessions, giving patients an opportunity to assemble relevant medical information and ensuring privacy when completing sensitive questions,^11^, ^12^ such as past medical history, cancer risk factors, sexual orientation, and gender identity.

Despite the benefits of an electronic approach, important questions remain about the representativeness of self‐reported data, particularly through the lens of diversity and equity, given the potential risk of perpetuating disparities in access to high‐quality oncology care through the deployment of digital tools. Previous research has shown disparities in access to patient portals by race, ethnicity, age, sex, and English proficiency.^13^, ^14^, ^15^, ^16^ This study aimed to describe patient portal usage and EPQ completion at a high‐volume comprehensive cancer center and to identify differences by patient clinical characteristics and sociodemographic variables.

## 2 METHODS

### 2.1 Study population and study design

Moffitt serves a large primary catchment area composed of 15 Florida counties encompassing 30% of the state's population,^17^ and serves patients from all 50 states and more than 140 countries.^18^ Patients seek care at Moffitt for services across the cancer continuum, including prevention, screening, diagnosis, treatment, survivorship care, second opinions, and therapy for select benign or premalignant conditions. To better understand the characteristics of patients who complete or do not complete the EPQ and assess the generalizability of these self‐reported data for research, we conducted a comprehensive analysis of all new adult patients from 2009 to 2020.

### 2.2 Data collection

Data were obtained via various data fields from electronic data capture systems, with different data types available for different years, as described below.

### 2.3 Patient‐reported data

In 2009, Moffitt sought to convert existing paper‐based NP intake forms into a single EPQ administered to all NPs. To harmonize existing paper‐based forms developed by individual clinics, a multidisciplinary team of clinical faculty, staff, and researchers was convened and has governed EPQ content to this day. Questions were included in the EPQ only if there was clear clinical utility. The specific formatting of questions was determined based on existing standards and potential downstream uses of the data, including clinical decision support, quality improvement, and research. Moffitt's Patient Family Advisory Council was consulted during the EPQ revision process.

The EPQ was designed to be completed by patients once, as a baseline patient intake form, and includes 12 core modules with questions pertaining to demographics, visit history, personal and family history of cancer, other family history, women's reproductive history, cancer risk factors, past medical history, current symptoms, surgical history, psychosocial assessment, and quality of life (Table S1). Based on different skip patterns, patients who received the core EPQ may answer anywhere between 87 and 230 questions. In addition to these core EPQ modules, disease‐specific “spokes” include questions uniquely relevant to subsets of cancer patients, such as questions pertaining to the diseases themselves, relevant risk factors, and quality of life considerations; these disease‐specific questions were interspersed with “core” questions to optimize question flow. Spokes are currently used for patients with thoracic, cutaneous, prostate, bladder, colorectal, pancreas, liver, malignant hematology, ovarian or breast cancers. The EPQ incorporated previously validated tools to examine patient cancer risk, physical health, and quality of life. Examples of such tools include the International Physical Activity Questionnaires, Fagerstorm Test for Nicotine Dependence, Semiquantitative Food Frequency Questionnaire, and Short Form 12 (SF‐12) Health Survey. To enhance the quality of clinical care and research for sexual and gender minority populations, questions to ascertain patients' sexual orientation and gender identity (SOGI) were added to the EPQ in 2016. Questions, associated definitions, and response options were modeled on the Fenway Institute's “Do Ask, Do Tell” guidelines.^19^ The questionnaire has historically been available in English with shorter, paper versions available in Spanish; development of a Spanish‐language EPQ is underway.

To optimize accessibility, the EPQ was integrated into Moffitt's electronic patient portal. Once a patient is scheduled for a NP appointment a link to register for the portal and complete the EPQ is emailed to the patient, a process which has been consistently implemented over time. Patients who have not completed the EPQ at the time of their initial appointment are asked to complete it on a tablet in the clinic, although only a small percentage of patients use the tablet (12.8% in 2022).

### 2.4 Clinic appointments and patient portal utilization

The Soarian scheduling system, a Cerner product, was used to identify the number of patients with NP appointments, select demographic characteristics as recorded by scheduling staff, preferred language, and the subset who accessed the patient portal at least once prior to the date of the NP appointment. This information was available starting in 2014.

### 2.5 Cancer characteristics

The Moffitt Cancer Registry is a highly curated database used to record all malignancies diagnosed and/or treated at Moffitt that are defined as reportable to Florida's statewide cancer registry.^20^ Certified tumor registrars and abstractors document information relevant for each cancer diagnosis. In this analysis, we examined EPQ completion by demographic/clinical characteristics among all NPs to Moffitt, as well as within the subset who were included in the Cancer Registry. Variables extracted from the Cancer Registry from 2009 to 2020 included: sex, age at presentation/diagnosis, race/ethnicity, primary cancer site, and stage at diagnosis/presentation.

### 2.6 Statistical analysis

Numbers of patients scheduled for NP appointments were tabulated by year of visit and demographic characteristics, as were the numbers and percentages of those who accessed the patient portal. Similarly, the number of patients who completed the EPQ were tabulated by year, as were the subset of those patients entered in the Cancer Registry with a date of first contact within 1 year of EPQ completion. Cancer Registry‐recorded demographics were described for patients who completed the EPQ, overall and among the subset of patients entered in the Cancer Registry within 1 year of their EPQ completion; chi‐squared and Cochran–Armitage tests were used to test statistical significance of observed differences.

Demographic and clinical characteristics for all NPs entered into the Cancer Registry were described. Patient characteristics were described separately among those who did and did not complete the EPQ. Unadjusted logistic regression was used to estimate the association between the odds of completing the EPQ and each of the described variables. Multivariable logistic regression was used to control for potential confounding. Associations between EPQ completion and stage at presentation and stage at diagnosis were adjusted for all demographic characteristics. Multivariable logistic models were used to examine possible interactions between tumor stage and each of the demographic factors including race, ethnicity, and language preference. Percentages of patients who completed and did not complete the EPQ were presented by cancer characteristics. Using data captured in 2016–2020, sex assigned at birth, relationship status, gender identity, and sexual orientation were described for all patients who completed EPQ, and separately, among those with a Cancer Registry‐recorded date of first contact within 1 year of EPQ completion. Lastly, among patients in the Cancer Registry, logistic regression was used to examine the association between demographics and whether a patient had complete data for questions on SOGI.

De‐identified data were used and therefore institutional review board approval was not required. Analysis were conducted using R version 4.02 (RRID:SCR_001905)^21^ with tidyverse (RRID:SCR_019186)^22^ and DescTools.^23^ Two sided *p* ≤ 0.05 were considered statistically significant.

## 3 RESULTS

### 3.1 Patient portal use and EPQ completion

Patient portal usage increased over time from 76.3% in 2014 to 83.3% in 2020 (Table 1). The number of patients with NP appointments completing the EPQ increased from 743 patients in 2009 to over 17,000 in 2019 and over 15,000 patients in 2020. EPQ completion rates steadily increased from 71.1% in 2014, peaked at 84.2% in 2017, and decreased to 77.7% in 2020, the first year of the COVID‐19 pandemic. During 2014–2020, 51% of patients who completed the EPQ were recorded in the Cancer Registry as having been diagnosed and/or treated for cancer within 1 year of EPQ completion. The remaining patients likely received other services at Moffitt, such as second opinions, cancer prevention and screening, and treatment for nonmalignant conditions.

**TABLE 1:** Patient portal usage and electronic patient questionnaire (EPQ) completion among patients with a new patient (NP) appointment at Moffitt in 2009–2020.

	Scheduling	Portal usage		EPQ completion		EPQ completion and new diagnosis	
Patient characteristics^a^	Number of patients with NP appointment	Number of patients with NP appointments and used portal by the time of the NP appointment		Number of patients who completed EPQ		Number of patients who completed EPQ and had a date of first contact within 1 year of EPQ completion	
	*n*	*n*	%^b^	*n*	%^c^	*n*	%^d^
Year				EPQ completion year			
2009				743		211	28.4
2010				8605		3791	44.1
2011				11,641		5425	46.6
2012				10,030		4951	49.4
2013				11,198		5686	50.8
2014	16,388	12,496	76.3	11,658	71.1	5832	50.0
2015	16,466	12,876	78.2	12,682	77.0	6400	50.5
2016	18,482	14,649	79.3	14,724	79.7	7541	51.2
2017	19,073	15,798	82.8	16,065	84.2	8586	53.4
2018	20,594	16,799	81.6	16,543	80.3	8450	51.1
2019	20,896	17,135	82.0	17,043	81.6	8728	51.2
2020	19,585	16,322	83.3	15,210	77.7	7503	49.3
From 2014 to 2020	131,484	106,075	80.7	103,925	79.0	53,040	51.0
All years total				146,142		73,104	50.0

^a^ The Soarian Scheduling System was used as the data source for this table.

^b^ Percentage calculated as the number of unique patients with NP appointments and used portal by the time of the NP appointment out of the Number of unique patients with NP appointments.

^c^ Percentage calculated as the number of unique patients who completed the EPQ out of the number of patients with NP appointments.

^d^ Percentage calculated as the number of unique patients who completed the EPQ and were entered in Cancer Registry within 1 year of EPQ completion out of the number of patients who completed the EPQ.

Of the approximately 131,000 patients who were scheduled for NP appointments in 2014–2020, 54% were female, 56% were aged ≥65 years, 86% were White, and 89% were non‐Hispanic (Table 2). Statistically significant differences of small magnitude were observed in the proportions of patients with NP appointments who accessed the patient portal across categories of sex and age. Patient portal usage varied by race (82%, 67%, 81%, and 71% for patients of White, Black, Asian, and Other races, respectively, *p* < 0.0001) and ethnicity (70% and 82% for Hispanic and non‐Hispanic patients, respectively, *p* < 0.0001). EPQ completion rates were >95% for all age groups within the age range of 18–55 years and decreased to 88% and 68% for patients ages 55–64 and ≥65 years, respectively. Higher EPQ completion rates were observed for White and Asian patients as compared with Black and Other races. Similarly, regarding ethnicity, 81% of non‐Hispanic patients completed the EPQ compared to 68% of Hispanic patients. The percentage of EPQ completers who were diagnosed and/or treated for cancer within 1 year of EPQ completion was greater for males (57% male vs. 46% female, *p* < 0.0001) and older patients (57% of patients aged ≥65 vs. 46% aged 18–64, *p*‐trend < 0.0001).

**TABLE 2:** Patient characteristics, portal usage and electronic patient questionnaire (EPQ) completion among patients with a new patient (NP) appointment at Moffitt in 2014–2020.

	Scheduling	Portal usage		EPQ completion		EPQ completion and new diagnosis	
Patient characteristics^a^	Number of patients with NP appointment	Number of patients with NP appointments and used portal by the time of the NP appointment		Number of patients who completed EPQ		Number of patients who completed EPQ and had a date of first contact within 1 year of EPQ completion	
	*n*	*n*	%^b^	*n*	%^c^	*n*	%^d^
Sex							
Female	70,516	57,501	81.5	56,928	80.7	26,255	46.1
Male	60,968	48,574	79.7	46,992	77.1	26,784	57.0
Missing				5		1	20.0
Total	131,484	106,075	80.7	103,925	79.0	53,040	51.0
*p*‐value^e^		<0.0001				<0.0001	
Age							
18–25	1644	1223	74.4	1577	95.9	456	28.9
26–35	4651	3789	81.5	4610	99.1	1458	31.6
36–45	9131	7515	82.3	8691	95.2	3154	36.3
46–55	16,654	13,619	81.8	15,936	95.7	7380	46.3
56–64	25,684	20,969	81.6	22,665	88.2	12,035	53.1
≥65	73,720	58,960	80.0	50,446	68.4	28,557	56.6
Total	131,484	106,075	80.7	103,925	79.0	53,040	51.0
*p*‐value^f^		<0.0001				<0.0001	
Race^g^							
American Indian, Aleutian, or Eskimo	266	200	75.2	207	77.8	100	48.3
Asian	2230	1796	80.5	1751	78.5	826	47.2
Black/African American	8049	5357	66.6	5460	67.8	2665	48.8
Native Hawaiian or Other Pacific Islander	203	153	75.4	132	65.0	61	46.2
White	112,562	92,653	82.3	89,062	79.1	46,929	52.7
Other	4238	2916	68.8	2717	64.1	1346	49.5
More than one race	640	502	78.4	470	73.4	236	50.2
Missing	3296	2498	75.8	4126	125.2	877	21.3
Total	131,484	106,075	80.7	103,925	79.0	53,040	51.0
*p*‐value^e^		< 0.0001^h^				< 0.0001^h^	
Ethnicity							
Hispanic	11,331	7980	70.4	7670	67.7	3569	46.5
Multiple	25	22	88.0				
Non‐Hispanic	116,643	95,366	81.8	94,086	80.7	48,448	51.5
Missing	3485	2707	77.7	2169	62.2	1023	47.2
Total	131,484	106,075	80.7	103,925	79.0	53,040	51.0
*p*‐value^e^		<0.0001^i^				<0.0001^i^	

^a^ The Soarian scheduling system was used as the data source for this table.

^b^ Percentage calculated as the number of unique patients with NP appointments and used portal by the time of the NP appointment out of the Number of unique patients with NP appointments.

^c^ Percentage calculated as the number of unique patients who completed the EPQ out of the number of patients with NP appointments.

^d^ Percentage calculated as the number of unique patients who completed the EPQ and were entered in Cancer Registry within 1 year of EPQ completion out of the number of patients who completed the EPQ.

^e^ *p*‐Values were calculated using chi‐squared tests for sex, race, and ethnicity with missing data excluded from the analysis.

^f^ *p*‐values were calculated using the Cochran–Armitage trend test for age with missing data excluded from the analysis.

^g^ Portal data race categorizations differ from those used in the Moffit Cancer Registry. Thus, the following race subcategories were combined as Asian: Asian Indian, Pakistani, Chinese, Filipino, Japanese, Kampuchean (including Khmer, Cambodian), Korean, Laotian, Hmong, Melanesian, Other Asian including Asian and Oriental, Thai, and Vietnamese; Native Hawaiian or Other Pacific Islander: Guamanian, Hawaiian, Samoan, Chamorran, Fiji Islander, Tahitian, Tongan, New Guinean, Micronesian, Polynesian, and Pacific Islander; and White: White and White Hispanic.

^h^ For sample size considerations, when analyzing race, American Indian, Aleutian, Eskimo, native Hawaiian or other Pacific islander, other race, or multiple races were combined as one category to compare with Asian, Black, and White.

^i^ When analyzing ethnicity, multiple ethnicities were combined with Hispanic to compare with non‐Hispanic.

### 3.2 Demographic and cancer characteristics associated with EPQ completion

Table 3 presents associations between demographics and EPQ completion among NPs in 2009–2020 who were diagnosed and/or treated for cancer that same year. Females were more likely to complete the EPQ than males (odds ratio [OR] = 1.17, 95% confidence interval [CI] = 1.14–1.20). EPQ completion rates were lower among those ages 18–25 (OR = 0.70, 95% CI = 0.52–0.93) and higher among those ages 56–64 (OR = 1.16, 95% CI = 1.09–1.23) as compared to those aged ≥65 (Table 3). Asian patients were not statistically different from White patients with respect to EPQ completion. However, compared to White patients, Black or African American patients (OR = 0.59, 95% CI = 0.56–0.63) and patients categorized as other race (OR = 0.56, 95% CI = 0.52–0.61) were less likely to complete the EPQ. Similarly, Hispanic patients were less likely to complete the EPQ than non‐Hispanic patients, although this difference was not statistically significant in the multivariable model that included language preference, where patients who prefer Spanish (OR = 0.40, 95% CI = 0.36–0.44) or other languages (OR = 0.60, 95% CI = 0.50–0.71) had significantly lower EPQ completion rate as compared to patients who prefer English. Notably, a minimal proportion (0.2%) of patients identified as being both Hispanic and Black.

**TABLE 3:** Electronic Patient Questionnaire (EPQ) completion by patient demographic characteristics for new patients to Moffitt who received a cancer diagnosis between 2009 and 2020.

Demographic characteristics^a^	Total	EPQ non‐completers^b^		EPQ completers^b^		Crude odds ratios (95% confidence interval)	Multivariable odds ratio (95% confidence interval)
	*n*	*n*	%	*n*	%		
Sex							
Female	53,575	15,775	29.4	37,800	70.6	1.18 (1.15–1.21)	1.17 (1.14–1.20)
Male	56,228	18,522	32.9	37,706	67.1	1.00 (ref.)	1.00 (ref.)
Other	13	1	7.7	12	92.3		
Age at presentation							
18–25	1054	387	36.7	667	63.3	0.86 (0.76–0.98)	1.38 (1.01–1.89)
26–35	3213	920	28.6	2293	71.4	1.25 (1.15–1.35)	1.45 (1.18–1.78)
36–45	6879	1918	27.9	4961	72.1	1.29 (1.22–1.37)	1.33 (1.16–1.53)
46–55	15,999	4556	28.5	11,443	71.5	1.26 (1.21–1.30)	1.27 (1.16–1.39)
56–64	25,081	7327	29.2	17,754	70.8	1.21 (1.17–1.25)	1.10 (1.03–1.17)
≥65	57,590	19,190	33.3	38,400	66.7	1.00 (ref.)	1.00 (ref.)
Age at diagnosis							
18–25	1398	508	36.3	890	63.7	0.88 (0.79–0.99)	0.70 (0.52–0.93)
26–35	3703	1057	28.5	2646	71.5	1.26 (1.18–1.36)	0.98 (0.81–1.19)
36–45	7978	2229	27.9	5749	72.1	1.30 (1.24–1.37)	1.07 (0.94–1.22)
46–55	18,430	5373	29.2	13,057	70.8	1.23 (1.18–1.27)	1.04 (0.96–1.14)
56–64	26,741	7825	29.3	18,916	70.7	1.22 (1.18–1.26)	1.16 (1.09–1.23)
65+	51,566	17,306	33.6	34,260	66.4	1.00 (ref.)	1.00 (ref.)
Race							
White	97,646	29,227	29.9	68,419	70.1	1.00 (ref.)	1.00 (ref.)
Black/African American	6541	2603	39.8	3938	60.2	0.65 (0.61–0.68)	0.59 (0.56–0.63)
Asian Indian, Pakistani	394	106	26.9	288	73.1	1.06 (0.96–1.18)^c^	1.06 (0.95–1.17)^c^
Other Asian including Asian and Oriental	1503	438	29.1	1065	70.9		
American Indian or Alaska Native	267	91	34.1	176	65.9	0.47 (0.44–0.51)^d^	0.56 (0.52–0.61)^d^
Native Hawaiian or Other Pacific Islander	168	68	40.5	100	59.5		
Other	2521	1248	49.5	1273	50.5		
Missing	776	517	66.6	259	33.4		
Ethnicity							
Non‐Hispanic	100,448	30,363	30.2	70,085	69.8	1.00 (ref.)	1.00 (ref.)
Hispanic	8739	3439	39.4	5300	60.6	0.67 (0.64–0.70)	0.96 (0.91–1.02)
Missing	629	496	78.9	133	21.1		
Language preference							
English	105,989	32,292	30.5	73,697	69.5	1.00 (ref.)	1.00 (ref.)
Spanish	3162	1716	54.3	1446	45.7	0.37 (0.34–0.40)	0.40 (0.36–0.44)
Other languages^e^	578	248	42.9	330	57.1	0.58 (0.49–0.69)	0.60 (0.50–0.71)
Missing	87	42	48.3	45	51.7		

^a^ The MCC Cancer Registry was used as the data source for this table.

^b^ Patients in this table were restricted to those whose date of first contact were between 2009 and 2020.

^c^ Asian Indian, Pakistani, and other Asian were grouped as Asian to compare with White in the logistic regression analysis.

^d^ American Indian or Alaska, native Hawaiian, and other were grouped as other race to compare with White in the logistic regression analysis.

^e^ The top 10 other languages preferred by the patients were: Arabic, Vietnamese, Creole, Russian, Polish, Portuguese, Mandarin, sign language, Korean, and Albanian.

**FIGURE 1:**
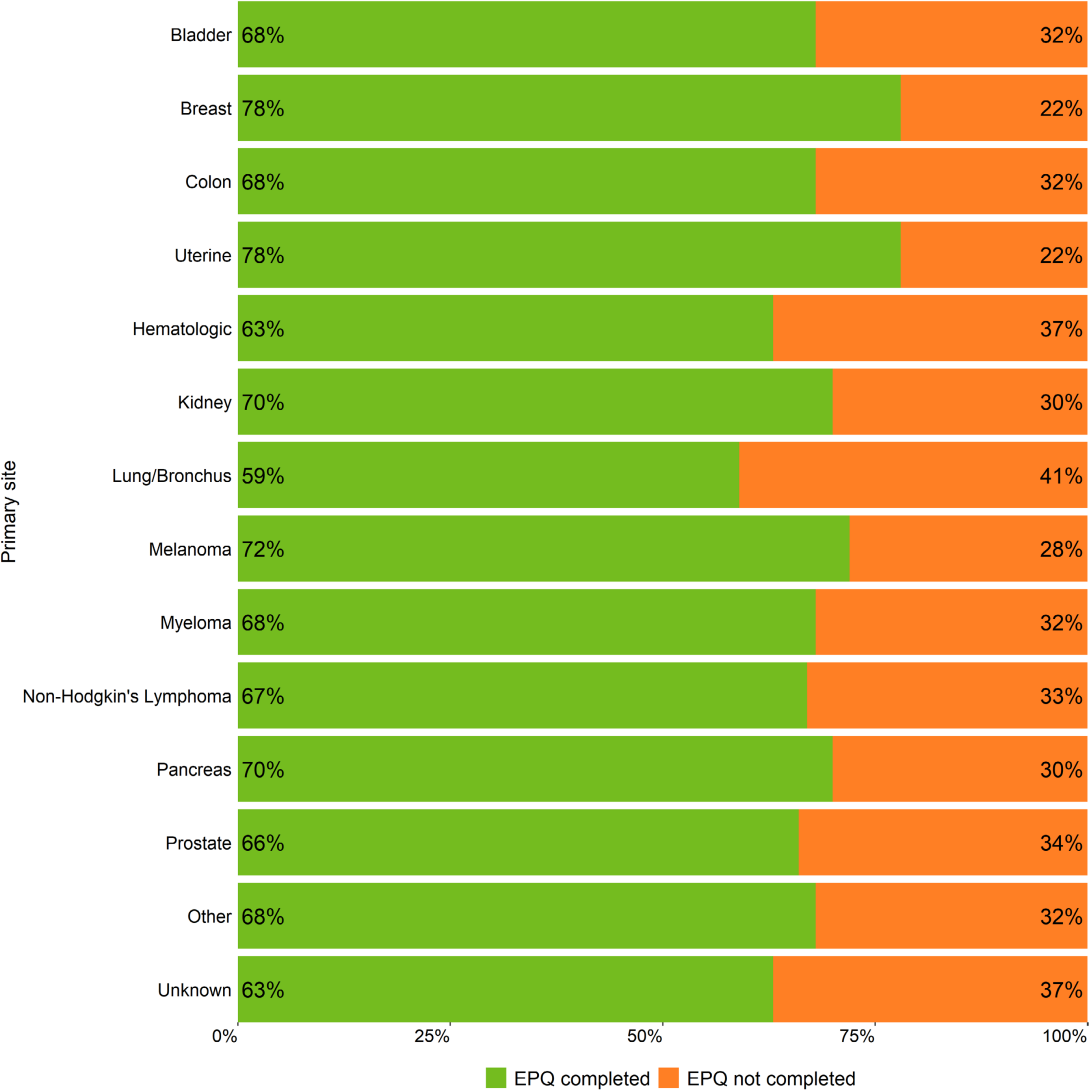
Electronic Patient Questionnaire (EPQ) completion by primary site of the cancer for new patients to Moffitt who received a cancer diagnosis between 2009 and 2020. The bar graph depicts the percent of patients who completed (green) and did not complete the EPQ (orange) by the primary site of the cancer among patients who received a new cancer diagnosis between 2009 and 2020. Sites that represented less than 2% of the total sample size were collapsed into the “Other” category.

**FIGURE 2:**
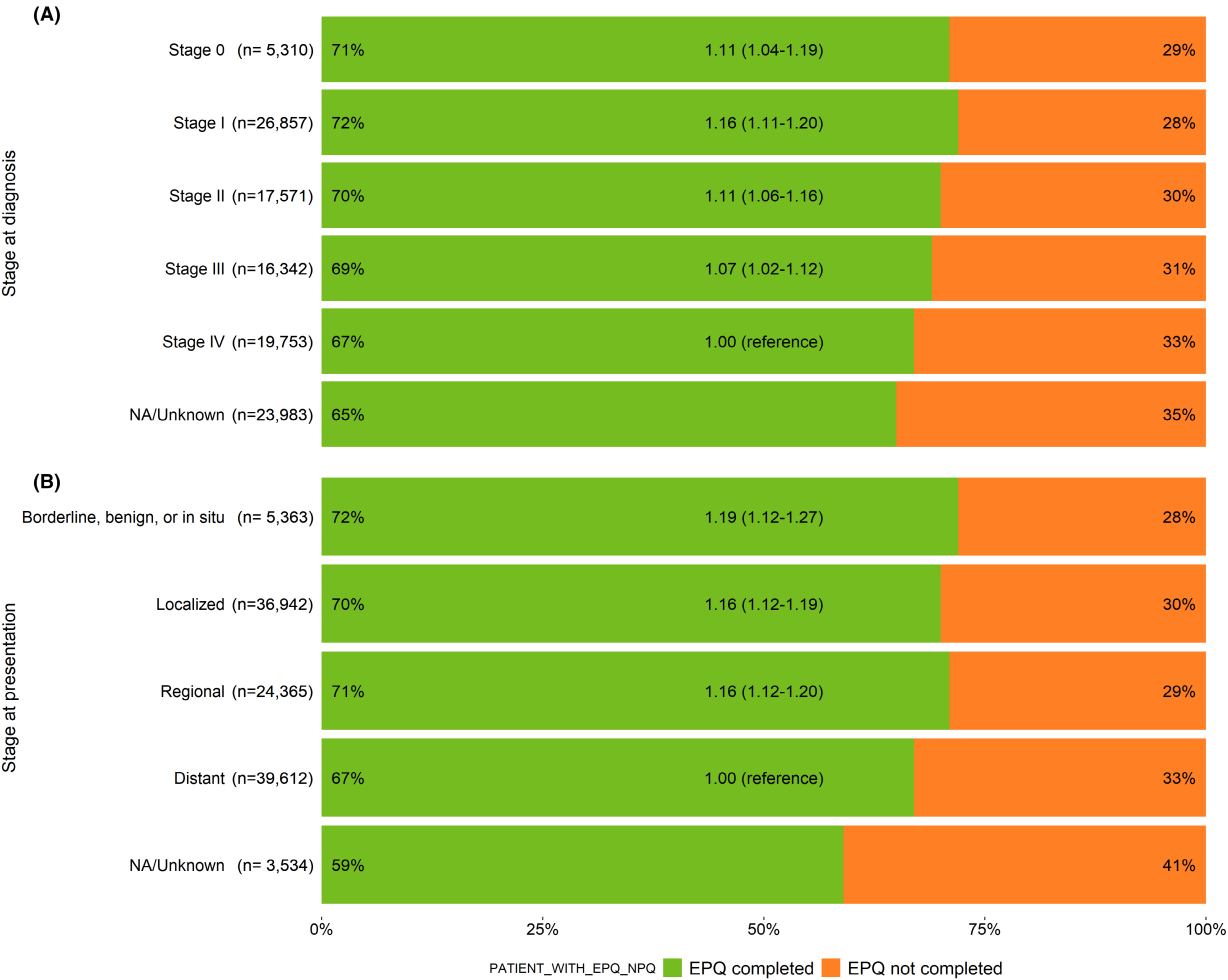
Electronic Patient Questionnaire (EPQ) completion by stage of the cancer for new patients to Moffitt who received a cancer diagnosis between 2009 and 2020. The percent of patients who completed (green) and did not complete the EPQ (orange) by (A) stage at diagnosis and (B) stage at presentation among patients who received a new cancer diagnosis between 2009 and 2020. The odds ratios presented were adjusted for sex, age at presentation, age at diagnosis, race, and ethnicity.

EPQ completion rates ranged from 59% of patients with lung cancer to 78% of patients with breast cancer or uterine cancer (Figure 1). Compared to patients with distant disease at the time of presentation to Moffitt, the EPQ was more often completed by patients with less advanced disease (Figure 2). For example, patients presented at Moffitt with a localized (OR = 1.16, 95% CI = 1.12–1.19) or a regional disease (OR = 1.16, 95% CI = 1.12–1.20) were 16% more likely to complete the survey than those who had metastatic disease. No statistically significant interaction effects were found between stage at presentation and race, ethnicity, or language preference, respectively (*p*‐interaction = 0.162–0.894). Similar results were observed for stage at diagnosis (Figure 2).

### 3.3 Sexual orientation, gender identity, and relationship status

Self‐reported sex at birth and relationship status among the 145,486 patients completing EPQ in 2009–2020 as well as gender identity and sexual orientation among the 69,540 patients completing EPQ in 2016–2020 are described in Table 4. Fifty‐four percent of EPQ respondents reported female sex at birth, 44% reported male, 4 reported “other” and 67 declined to answer. Using data collected after 2016, gender identity was reported as follows: 54.5% female, 45.2% male, 16 female‐to‐male transgender, 8 male‐to‐female transgender, 5 genderqueer, 15 identified with another gender category, and 89 declined to answer. Regarding sexual orientation, 1298 (2%) of EPQ respondents reported being lesbian, gay, or homosexual, 298 (0.4%) reported being bisexual, 360 (0.5%) reported “something else”, and 259 (0.4%) responded “don’t know”. Notably, patients who were male, of younger age, and racial minorities were more likely to complete questions on SOGI than patients who were female, of older age, and White, respectively (Table S2). Most EPQ respondents (66%) reported being married, followed by 11% single, and 11% divorced or separated. Among the subset of EPQ respondents who were diagnosed and/or treated for cancer that same year, sex at birth and gender identity were more evenly distributed across males and females than for EPQ respondents overall, with other gender identities comprising approximately 0.1% of both groups. Sexual orientation and relationship status were similarly distributed among all EPQ respondents and the subset in the cancer registry.

**TABLE 4:** Electronic Patient Questionnaire (EPQ) completion by patient sexual orientation and gender identity (SOGI) for new patients (NPs) to Moffitt between 2009 and 2020.

EPQ SOGI data (available years)	All Moffitt patients who completed an EPQ^a^		EPQ completers newly diagnosed and/or treated for cancer at Moffitt within 1 year of EPQ completion^b^	
Variables	*n*	%	*n*	%
Sex at birth (2009–2020)				
Female	79,136	54.4	35,888	49.2
Male	64,510	44.3	36,270	49.7
Other	4	0.0	1	0.0
Declined	67	0.0	21	0.0
Missing	1769	1.2	738	1.0
Marital/relationship status (2009–2020)				
Single	16,560	11.4	7327	10.0
Domestic partnership	4435	3.0	2180	3.0
Married	96,151	66.1	49,030	67.2
Divorced or separated	15,292	10.5	7588	10.4
Widowed	10,402	7.1	5670	7.8
Declined	892	0.6	390	0.5
Missing	1754	1.2	733	1.0
Gender identity (2016–2020)				
Female	37,920	54.5	17,664	49.1
Male	31,440	45.2	18,215	50.7
Transgender, female to male	16	0.0	5	0.0
Transgender, male to female	8	0.0	4	0.0
Genderqueer	5	0.0	0	0.0
Additional gender category	15	0.0	4	0.0
Declined	89	0.1	34	0.1
Missing	47	0.1	19	0.1
Sexual orientation (2016–2020)				
Straight or heterosexual	59,053	84.9	30,573	85.1
Lesbian, gay, or homosexual	1298	1.9	599	1.7
Bisexual	298	0.4	111	0.3
Something else	360	0.5	169	0.5
Do not know	259	0.4	121	0.3
Missing	8272	11.9	4372	12.2

^a^ Includes all NPs to Moffitt who completed an EPQ in 2009–2020, including screening patients, second opinions, those being treated for premalignant or nonmalignant conditions, as well as patients diagnosed with and/or treated for cancer.

^b^ Includes the subset of NPs to Moffitt who completed an EPQ and for whom a date of first contact as recorded in the cancer registry was within 1 year of EPQ completion (i.e., new Moffitt patients who were diagnosed and/or treated for a reportable malignancy the same year as EPQ completion).

## 4 DISCUSSION

Moffitt patient portal usage and EPQ completion rates have been consistently high among NPs seen at Moffitt since 2014, averaging 81% and 79%, respectively, notably higher than rates historically reported by others,^7^, ^24^ although consistent with more recent portal usage that has increased with the expansion of telehealth following the COVID‐19 pandemic.^25^, ^26^, ^27^, ^28^ The Moffitt patient portal provides patients with access to several services relevant to clinical care (e.g., scheduling) and encourages advanced completion of clinically relevant patient forms such as the EPQ, which may contribute to high completion rates. In addition, the availability of the EPQ via tablet in the clinic also serves to optimize completion rates,^7^ particularly among patients accessing emergent care.

We aimed to assess the possibility that downstream analyses could be prone to selection bias and to identify subgroups to target in future efforts to encourage completion. Although absolute differences in completion rates across demographic categories were minimal (5%–10%), these differences reached statistical significance, likely due in part to the large samples sizes. Nevertheless, attention is warranted regarding the lower completion rates observed in males versus females, older ages versus younger ages, Black/African American, and other non‐White/non‐Asian races versus White and Asian races, and patients whose preferred language is Spanish or another language other than English.^29^ Translation of the EPQ to other languages may improve EPQ completions rates and provide additional benefits, including improved data quality and patient experience.^30^, ^31^, ^32^ Future research should implement and test additional targeted strategies to improve accessibility of portals and validated questionnaires in patients' native languages and targeted outreach to racial/ethnic minority patients to address technical, language, and access barriers.^30^, ^31^

Differences in EPQ completion were observed for primary cancer site, however, the overall number of questions did not vary greatly across disease spokes. Patients with more extensive disease, including those with worse symptoms, may be less likely to complete a lengthy questionnaire. For example, patients with metastatic disease had lower completion rates than those with less advanced disease which may be due in part to the physical and/or psychosocial burdens associated with advanced cancer and the treatment side effects. Thus, future quality improvement efforts should seek to minimize patient burden, taking into account unique needs of patients with advanced disease and tailoring content accordingly, with critical input from patients.

The EPQ provides a unique opportunity for patients to discreetly share their SOGI with their oncology providers. Of the 69,540 NPs who completed the EPQ in 2016–2020, 1596 (2.3%) self‐identified as being lesbian, gay, or bisexual and 44 (<0.1%) self‐identified as transgender, genderqueer, or an additional gender category. These proportions are lower than estimates for the general population.^33^ This may be due to the older age of cancer patients compared to the general population, as a U.S. nationwide survey indicated that the percentage of the population identifying as a sexual orientation or gender identity minority is 7.1% overall, including 2.6% of individuals ages 58–72, and 0.8% of those over age 72.^33^ Another explanation may be that some patients chose not to disclose their sexual orientation or gender identity. Based on our analysis, these patients are more likely to be female, of older age, and racial minorities. Future research should assess sexual orientation and gender minority individuals' preferences for self‐identifying to healthcare providers and revise data collection instruments accordingly.

Moffitt was an early adopter of portal technology for patient reported information capture (2009) and collection of self‐reported data on sexual and gender identity (2016), well ahead of the goals set by the Centers for Medicare and Medicaid Services' Promoting Interoperability Program.^34^, ^35^ As a result, the data included in the current analysis span over a decade and include more than 100,000 patients, representing a major study strength. Some limitations should also be noted. Scheduling data were not available prior to 2014 at which time there was a change in the scheduling systems, and limited clinical information was available for patients not entered into the Cancer Registry; therefore, portal utilization and EPQ response could not be examined as functions of non‐cancer diagnoses and/or procedures such as screening exams. Since EPQ data are captured only at the time of clinical intake, current study findings may not be generalizable to the electronic acquisition of patient‐reported outcomes in the follow‐up setting. Finally, results of this analysis may not be directly generalizable to other healthcare institutions, although the overall approach is important and generalizable, in that healthcare organizations should be encouraged to evaluate their data acquisition methods across patient subgroups to identify potential disparities in data completeness.

In summary, rates of patient portal use and EPQ completion were robust over time and among subgroups of patients defined by demographic and clinical factors. Differences in EPQ completion between patient subgroups can provide important insights for process improvement impacting downstream use of the data including clinical care, quality improvement, and research projects. Future studies are needed to implement and test targeted strategies to increase access to and use of electronic questionnaires among racial/ethnic minority patients as well as patients with more advanced disease and to extend this work into the follow‐up setting. While digital tools can rapidly scale data collection in the clinical setting, it is important to minimize patient burden and maximize both clinical utility and patient satisfaction. Finally, in an era where real world data are increasingly being used in research and cancer care delivery, an intentional and purposeful examination of the representativeness of minority populations in those datasets is imperative to ensure predictive models trained on such data do not perpetuate existing healthcare disparities.

## AUTHOR CONTRIBUTIONS

**Dana E. Rollison:** Conceptualization (equal); methodology (equal); project administration (equal); supervision (equal); visualization (equal); writing – original draft (equal). **Brian D. Gonzalez:** Conceptualization (equal); methodology (equal); writing – original draft (equal). **Kea Turner:** Writing – review and editing (equal). **Heather S. L. Jim:** Writing – review and editing (equal). **Yayi Zhao:** Data curation (equal); formal analysis (equal); methodology (equal); validation (equal); visualization (equal); writing – original draft (equal). **Rossybelle P. Amorrortu:** Project administration (equal); validation (equal); writing – original draft (equal). **Rachel Howard:** Writing – review and editing (equal). **Kavita M. Ghia:** Data curation (equal); writing – review and editing (equal). **Bryan Ngo:** Writing – review and editing (equal). **Phillip Reisman:** Writing – review and editing (equal). **Colin Moore:** Writing – review and editing (equal). **Randa Perkins:** Writing – review and editing (equal). **Robert J. Keenan:** Writing – review and editing (equal). **David A. Sallman:** Writing – review and editing (equal). **Cristina M. Naso:** Writing – review and editing (equal). **Edmondo J. Robinson:** Writing – review and editing (equal). **Susan T. Vadaparampil:** Writing – review and editing (equal). **Vani N. Simmons:** Writing – review and editing (equal). **Matthew B. Schabath:** Writing – review and editing (equal). **Scott M. Gilbert:** Conceptualization (equal); project administration (equal); writing – review and editing (equal).

## FUNDING INFORMATION

This work was supported in part by the Collaborative Data Services Core at the H. Lee Moffitt Cancer Center & Research Institute, a comprehensive cancer center designated by the National Cancer Institute and funded in part by Moffitt's Cancer Center Support Grant (P30‐CA076292).

## CONFLICT OF INTEREST STATEMENT

Dana E. Rollison serves on the Board of Directors for NanoString Technologies, Inc. Brian D. Gonzalez reports fees unrelated to this work from Sure Med Compliance and Elly Health. Heather S.L. Jim previous consultant for RedHill Biopharma, Janssen Scientific Affairs, and Merck, current grant funding from Kite Pharma. David A. Sallman reports Consultancy/Advisory Board membership unrelated to this work for AbbVie, Agios, Aprea, Curis, Intellia, Kite, Magenta, Novartis, Syndax, Shattuck Labs & Takeda, and research funding from Jazz, Aprea. Matthew B. Schabath is an Associate Editor for Cancer Medicine. The following authors declare no conflicts of interests: Kea Turner, Yayi Zhao, Rossybelle P. Amorrortu, Rachel Howard, Kavita M. Ghia, Bryan Ngo, Phillip Reisman, Colin Moore, Randa Perkins, Robert J. Keenan, Susan T. Vadaparampil, Vani N. Simmons, Edmondo Robinson, and Scott M. Gilbert.

## ETHICS STATEMENT

De‐identified data were used in this analysis and therefore institutional review board approval was not required.

## Supporting information


Table S1.
Table S2.

## Data Availability

Datasets related to this article are available upon request from the corresponding author. All requests will require a proposal, outlining specific research questions, methods, and timelines for completion. Formal approval will be granted by the corresponding author based on the topic and ability to obtain appropriate ethics committee approval. Applicants will be required to complete a Data Use Agreement form prior to receiving any data.
