# Supplementary material for: Examining disparities in large‐scale patient‐reported data capture using digital tools among cancer patients at clinical intake
Source: Cancer Med. 2023 Aug 18;12(18):19033–46. doi: 10.1002/cam4.6459 (PMC10557830; doi:10.1002/cam4.6459)
Supplement: Supplementary file 1 — Table S1. Table S2. [file CAM4-12-19033-s001.docx]

**Supplementary Table 1**. Description of the modules and the number questions in the core Electronic Patient Questionnaire and in each disease-specific spoke.

| Questionnaire (core/spoke) | Module^a^ | Number of questions^b^ | | |
| --- | --- | --- | --- | --- |
|  |  | Total | Minimum | Maximum |
| Core EPQ |  |  |  |  |
|  | Demographics | 18 | 14 | 18 |
|  | Visit background | 4 | 2 | 4 |
|  | Personal history of cancer | 6 | 1 | 6 |
|  | Past medical history | 36 | 3 | 35^c^ |
|  | Current symptoms | 33 | 19 | 33 |
|  | Women's risk assessment^d^ | 24 | 4 | 24 |
|  | Surgical clinical history | 25 | 2 | 24^c^ |
|  | Family history of cancer | 11 | 5 | 11 |
|  | Other family history | 2 | 1 | 2 |
|  | Cancer risk assessment | 57 | 20 | 56^c^ |
|  | Psychosocial | 6 | 6 | 6 |
|  | Quality of life | 11 | 11 | 11 |
|  | Total | 233 | 87 | 230 |
| Breast spoke | Total | 239 | 88 | 235 |
| Cutaneous spoke | Total | 277 | 106 | 274 |
| Gastrointestinal spoke | Total | 259 | 97 | 256 |
| Genitourinary spoke | Total | 271 | 109 | 268 |
| Hematology spoke | Total | 270 | 115 | 267 |
| Ovarian spoke^c^ | Total | 241 | 91 | 238 |
| Thoracic spoke | Total | 269 | 100 | 266 |
| ^a^The core EPQ and disease-specific spokes contain the same list of modules. | | | |  |
| ^b^Questions may include sub-components. | |  |  |  |
| ^c^The maximum number of questions to be answered in the module is smaller than the total number of questions because certain questions are gender specific. | | | | |
| ^d^This applies to female patients only. | |  |  |  |

**Supplementary Table 2.** EPQ sexual orientation and gender identity data availability by patient demographic characteristics for new patients to Moffitt who completed EPQ versions 4 and 4.2 and were included in Cancer Registry between 2016-2020.

| Demographic characteristics^a^ | Total | Patients missing SOGI data^b^ | | Patients with  SOGI data^b^ | | Crude odds ratios (95% Confidence Interval) | Multivariable odds ratio (95% Confidence Interval) |
| --- | --- | --- | --- | --- | --- | --- | --- |
|  | n | n | % | n | % |  |  |
| Sex |  |  |  |  |  |  |  |
| Female | 17,842 | 2,521 | 14.1 | 15,321 | 85.9 | 0.79 (0.74-0.84) | 0.77 (0.72-0.82) |
| Male | 18,358 | 2,112 | 11.5 | 16,246 | 88.5 | 1.00 (ref.) | 1.00 (ref.) |
| Other | 7 | 0 | 0.0 | 7 | 100.0 |  |  |
|  |  |  |  |  |  |  |  |
| Age at presentation |  |  |  |  |  |  |  |
| 18 - 25 | 318 | 36 | 11.3 | 282 | 88.7 | 1.24 (0.89-1.79) | 1.31 (0.93-1.88) |
| 26 - 35 | 1,017 | 109 | 10.7 | 908 | 89.3 | 1.32 (1.09-1.63) | 1.45 (1.18-1.78) |
| 36 - 45 | 2,139 | 250 | 11.7 | 1,889 | 88.3 | 1.20 (1.05-1.38) | 1.33 (1.16-1.54) |
| 46 - 55 | 4,886 | 543 | 11.1 | 4,343 | 88.9 | 1.27 (1.15-1.40) | 1.36 (1.23-1.51) |
| 56 - 64 | 8,353 | 1,023 | 12.2 | 7,330 | 87.8 | 1.14 (1.05-1.23) | 1.18 (1.09-1.27) |
| >= 65 | 19,494 | 2,672 | 13.7 | 16,822 | 86.3 | 1.00 (ref.) | 1.00 (ref.) |
|  |  |  |  |  |  |  |  |
| Age at diagnosis |  |  |  |  |  |  |  |
| 18 - 25 | 419 | 52 | 12.4 | 367 | 87.6 | 1.13 (0.85-1.53) | Dropped |
| 26 - 35 | 1,151 | 127 | 11.0 | 1,024 | 89.0 | 1.29 (1.07-1.56) |  |
| 36 - 45 | 2,487 | 294 | 11.8 | 2,193 | 88.2 | 1.19 (1.05-1.36) |  |
| 46 - 55 | 5,746 | 652 | 11.3 | 5,094 | 88.7 | 1.25 (1.14-1.37) |  |
| 56 - 64 | 8,958 | 1,108 | 12.4 | 7,850 | 87.6 | 1.13 (1.05-1.22) |  |
| 65+ | 17,446 | 2,400 | 13.8 | 15,046 | 86.2 | 1.00 (ref.) | 1.00 (ref.) |
|  |  |  |  |  |  |  |  |
| Race |  |  |  |  |  |  |  |
| White | 32,736 | 4,079 | 12.5 | 28,657 | 87.5 | 1.00 (ref.) | 1.00 (ref.) |
| Black/African American | 1,997 | 310 | 15.5 | 1,687 | 84.5 | 0.77 (0.68-0.88) | 0.74 (0.66-0.85) |
| Asian Indian, Pakistani^c^ | 151 | 29 | 19.2 | 122 | 80.8 | 0.83 (0.67-1.03) | 0.81 (0.66-1.01) |
| Other Asian including Asian and Oriental | 544 | 73 | 13.4 | 471 | 86.6 |  |  |
| American Indian or Alaska Native^d^ | 92 | 11 | 12.0 | 81 | 88.0 | 0.66 (0.54-0.82) | 0.63 (0.51-0.78) |
| Native Hawaiian or Other Pacific Islander | 56 | 4 | 7.1 | 52 | 92.9 |  |  |
| Other | 466 | 94 | 20.2 | 372 | 79.8 |  |  |
| Missing | 165 | 33 | 20.0 | 132 | 80.0 |  |  |
|  |  |  |  |  |  |  |  |
| Ethnicity |  |  |  |  |  |  |  |
| Hispanic | 2,728 | 368 | 13.5 | 2,360 | 86.5 | 0.94 (0.84-1.05) | Dropped |
| Non-Hispanic | 33,452 | 4,258 | 12.7 | 29,194 | 87.3 | 1.00 (ref.) | 1.00 (ref.) |
| Missing | 27 | 7 | 25.9 | 20 | 74.1 |  |  |

^a^The MCC Cancer Registry was used as the data source for this table.

^b^Patients in this table were restricted to those who completed EPQ versions 4 and 4.2 between 2016-2020 and were entered in Cancer Registry.

^c^Asian Indian, Pakistani and other Asian were grouped as Asian, and ^d^American Indian or Alaska, native Hawaiian, and other were grouped as other race to compare with White in the logistic regression analysis.
